# *Inula Viscosa* Extract Inhibits Growth of Colorectal Cancer Cells *in vitro* and *in vivo* Through Induction of Apoptosis

**DOI:** 10.3389/fonc.2019.00227

**Published:** 2019-04-10

**Authors:** Rinat Bar-Shalom, Margalit Bergman, Shlomo Grossman, Naiel Azzam, Lital Sharvit, Fuad Fares

**Affiliations:** ^1^Department of Human Biology, Faculty of Natural Sciences, University of Haifa, Haifa, Israel; ^2^Faculty of Life Sciences, Bar-Ilan University, Ramat-Gan, Israel

**Keywords:** *Inula viscosa*, extract, colorectal cancer, apoptosis, caspases

## Abstract

Colorectal cancer (CRC) is the second most common cancer in females and the third in males worldwide. Conventional therapy of CRC is limited by severe side effects and by the development of resistance. Therefore, additional therapies are needed in order to combat the problem of selectivity and drug resistance in CRC patients. *Inula viscosa* (IV) is a well-known medicinal perennial herb in traditional medicine. It is used for different therapeutic purposes, such as; topical anti-inflammatic, diuretic, hemostatic, antiseptic, antiphlogistic, and in the treatment of diabetes. Several studies attempted to reveal the anti-cancer activity of different extracts prepared by different organic solvents from different parts of the IV plant. The aim of the present study is to examine the potential beneficial effects of IV leaf aqueous extract on the growth of colon cancer cells *in vitro* and *in vivo*. The results indicated that exposure of colorectal cancer cells to IV extract, significantly reduced cell viability in a dose and time dependent manner. Moreover, treatment of cells with 300 μg/ml of IV extract induced apoptosis, as it was detected by Annexin V/FITC/PI, TUNEL assay, and the activation of caspases. *In vivo* studies revealed that treatment with 150 or 300 mg/kg IV extract inhibited tumor growth in mice transplanted with MC38 cells. Tumors' weight and volume were significantly (*P* < 0.001) reduced when compared to untreated-control group. Staining of the paraffin section of tumors revealed that IV treatment inhibited cell proliferation and induced apoptosis. Additionally, no side effects such as; weight loss, behavior changes, ruffled fur or changes in kidney, and liver functions were observed. These results may indicate that active doses of IV extract are not toxic. Further studies are needed in order to identify the structure of the active compounds. Results from this study may contribute to the development of new and efficient strategies for treatment of human colon cancer.

## Introduction

Colorectal cancer (CRC) is the second most common cancer in females after breast cancer and the third most common cancer in males after lung and prostate cancers. In 2018, the global incidence of CRC in both sexes was ~1.85 million with 880,792 deaths worldwide (1). The first line of treatment for potentially curable CRC patients is surgery (2), which is performed in ~80% of patients, while half of them will experience a recurrence of the disease (3). Adjuvant therapy, such as chemotherapy, is administered in order to prevent local recurrence or distant metastases. In patients with metastasis, chemotherapy is the mainstay with the goal of prolonging survival and to maintain quality of life (2). The effectiveness of chemotherapy has been limited by severe side effects and by the development of resistance (4, 5). Therefore, conventional chemotherapy has no consistent benefit in overall survival. Additional therapies are therefore needed in order to combat the problem of selectivity and drug resistance in CRC patients. Natural products are considered to have anticancer activity with high effectiveness and less toxicity (6). More than 60% of drugs, currently in use for cancer treatment, have been isolated from natural products and numerous of them obtained from plant sources (7). In addition, due to the vast use in traditional medicine, there has been a growing interest in the use of medicinal herbs as a potential candidate for new anticancer therapeutic drugs.

**Figure 1:**
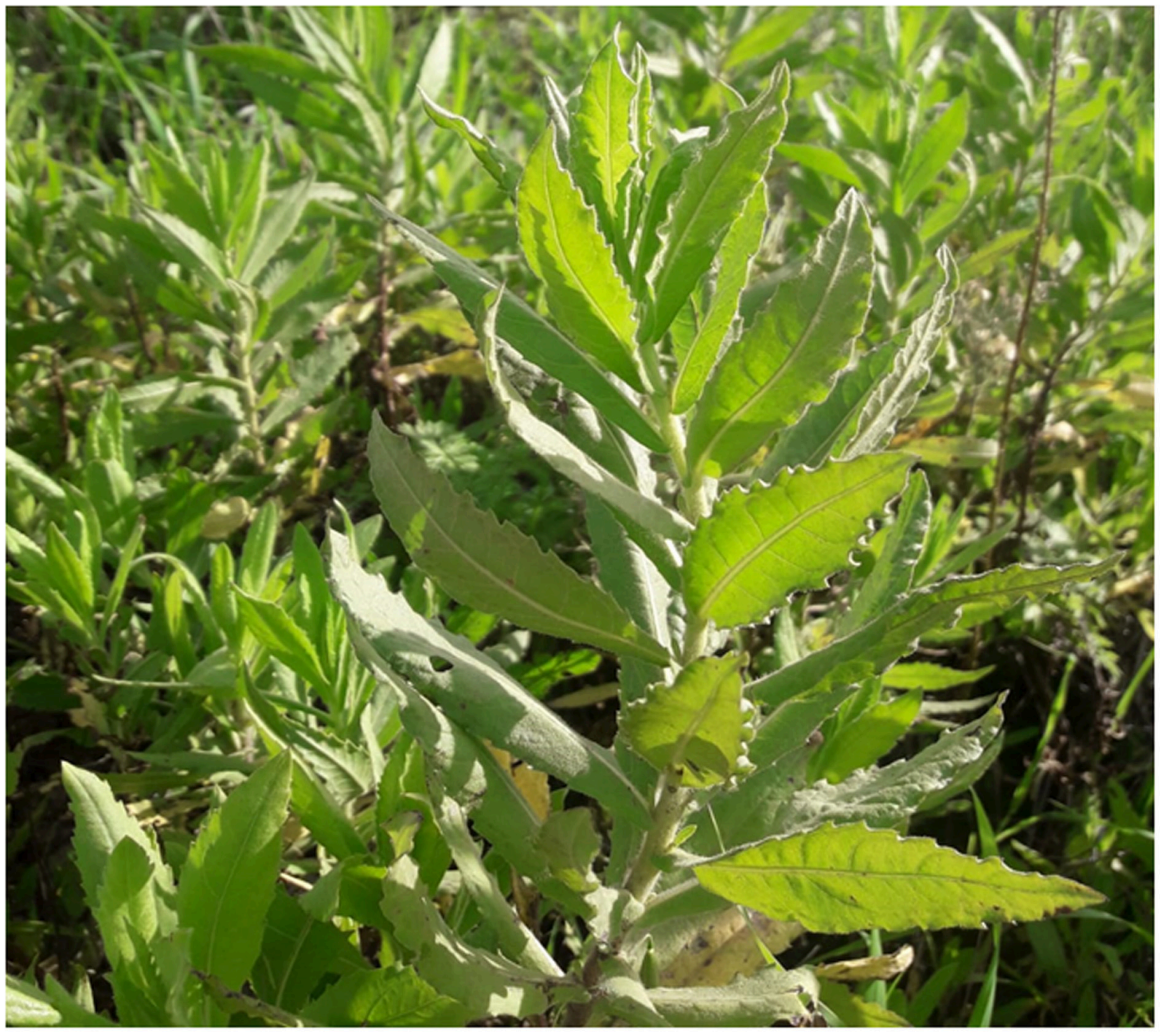
*Inula viscosa*. Photograph of the plant, *Inula viscosa*. The photograph was taken from a field at Bar-Ilan University, Ramat-Gan, Israel.

*Inula viscosa* (IV) *(L.)* Ait. (syn. *Dittrichia viscosa* Greuter) (Compositae) is a well-known medicinal perennial herb, native to the Mediterranean basin (Figure 1). It grows on hillslopes, damp habitats and roadsides (8). IV has sticky leaves with bright yellow flowers that bloom between August and November (9). In traditional medicine, IV is used as a remedy plant, that exhibits several medical uses such as; anti-inflammatory, antipyretic, and antimicrobial activity (10). Numerous studies have revealed the presence of different biologically active compounds in IV and their ability to induce apoptosis in cancer cells, including groups of phytochemicals such as polyphenols (11) and sesquiterpens (12). Among the polyphenols discovered, Danino et al. (9) isolated polyphenolic antioxidants from leaves of IV including seven derivatives from the caffeoylquinic acid (CQA) and dicaffeoylquinic acid (diCQA) family. There is a possibility for synergistic effects of these compounds in cancer treatment. This assumption, together with the need for novel therapeutic strategies of colon cancer, leads us to focus on investigating the anti-carcinogenic effects of IV leaf water extract on colon cancer cell growth *in vitro* and *in vivo*, and to elucidate the mechanism of its action. Therefore, the effects of IV extract on HCT116 and Colo320 cells of human origin colorectal cancer, were examined. Moreover, the potential beneficial effect of IV on tumor growth *in vivo* was evaluated using mice transplanted with MC38 cells that originated from mouse murine colon adenocarcinoma.

## Materials and Methods

### Crude Extract Preparation of *Inula viscosa*

Fresh leaves of IV were collected from a field at Bar-Ilan University, Israel. The leaves were sun-dried for 2 days and for another 4 days at room temperature. The dried leaves were homogenized in distilled water in a ratio of 1:8 (w/v). The homogenate was collected, filtered through Whathman No.1 filter paper and centrifuged at 20,000 x g for 10 min. The supernatant was frozen in liquid nitrogen and dried in a lyophilizer (0.07 mbar, −48°C). The resulting powdered extract was dissolved in water in order to obtain a 2% w/v solution.

### Cell Culture

Human colorectal cancer cell lines; HCT116 (well differentiated) and Colo320 (poorly differentiated) and the mouse murine adenocarcinoma cell line (MC38) were purchased from American Type of Culture Collection (Bethesda, MD, USA). Normal primary liver fibroblasts were purchased from Cell Biologics (Chicago, IL, USA). HCT116 and MC38 were cultured in Dulbecco's modified Eagle medium (DMEM; 4.5 g/L D-glucose) and Colo320 in RPMI-1640 medium. Normal primary liver fibroblasts were cultured in DMEM/F12 1:1 medium. Media were supplemented with 10% FBS, 1 mM L-glutamine, and 1% penicillin-streptomycin. Cells were cultured in a humidified incubator at 37°C with 5% CO_2_ saturation. The medium was changed every 3 days, and cells were passaged using Trypsin/EDTA. The cell lines were routinely tested for mycoplasma contamination with the Mycoplasma Test Kit EZ-PCR. All cell culture reagents, including Mycoplasma Test Kit, were supplied by Biological Industries (Kibbutz Beit Haemek, Israel).

### Cell Viability

Cell proliferation was determined using the sodium 3′-[1-(phenylaminocarbonyl)-3,4-tetrazolium]-bis (4-methoxy-6-nitro) benzene sulfonic acid hydrate (XTT) assay, according to the manufacturer's instructions (Biological Industries, Kibbutz Beit Haemek, Israel). In brief, cells were seeded in 100 μl of medium, using 96-well plates at a cell density of 104 cells/well. After 24 h, IV extract was added at different concentrations (100–300 μg/ml) and for varying time intervals (24–72 h). At least four independent experiments were performed each conducted in five replicates. Data are presented as the average proliferation percentage of the respective control. Based on the XTT experiments, IC50 values were calculated as the concentration of the plant extract that causes 50% inhibition in cell proliferation.

### Cytotoxicity Assay

The Lactate dehydrogenase (LDH) test was used to evaluate cytotoxicity of IV extract on colorectal cancer cells. LDH, a cytoplasmic enzyme, is rapidly released from the cells into the medium when the plasma membrane is damaged. The integrity of the plasma membrane following treatment was determined by measuring the LDH activity in the culture medium. Briefly, HCT116, Colo320, MC38, and normal primary liver fibroblasts cells were cultured in 96-well plates. IV extract was added in different concentrations. 24 h post treatment, the levels of LDH in the cell culture media were detected by the LDH Cytotoxicity Detection Kit ^PLUS^ (Roche, Mannheim, Germany) following the manufacturer's instructions.

### Cell Cycle Analysis

In order to study the effect of IV extract on cell cycle progression, DNA content analysis was performed by Fluorescence Activated Cell Sorter (FACS) of 10,000 cells stained with propidium iodide (PI) as described previously (13). In brief, cells were cultured in 25 cm^2^ flasks and treated with 300 μg/ml of IV extract for 14, 24, 48, or 72 h. At the time of analysis, cells were trypsinized, harvested, and centrifuged at 2,000 rpm for 5 min at 4°C. Cells were washed twice with cold PBS and then fixed in pre-chilled 70% ethanol at −20°C for 1 h. The cells were incubated with 0.1% NP-40 on ice for 5 min, and subsequently washed twice with cold PBS, each time by centrifugation at 2,000 rpm for 5 min at 4°C. Then, 1 ml of cold PBS containing RNase (100 μg/ml) was added to cells for 30 min. Finally, 50 μg/ml of PI was added to cells followed by incubation for 20 min on ice. DNA content was examined by flow cytometry using a FACSCantoII with FACSDiva software (Becton Dickenson, San Jose, CA, USA).

### Annexin V-FITC/PI Double-Staining Assay

Cell death was further analyzed by staining the cells with FITC-labeled Annexin V and propedium iodide (PI) using an Annexin V-FITC apoptosis detection kit (MBL, Nagoya, Japan), according to the manufacturer‘s instructions. Briefly, cells (2 × 10^5^) were seeded in 25 cm^2^ flasks and allowed to attach overnight. Cells were treated with 300 μg/ml of IV extract for 14, 24, 48, or 72 h. To detect early and late apoptosis, both adherent and floating cells were harvested together. Treated and untreated cells were harvested by trypsinization, washed, and suspended in ice-cold PBS. The washed cell pellet was suspended in ice-cold binding buffer containing FITC-conjugated Annexin V and PI. Samples were incubated in room temperature for 15 min in dark before analysis by FACSCantoII Flow cytometry (Becton Dickenson, San Jose, CA). The Annexin V-FITC-negative/PI-negative population was considered to include all normal healthy cells. Annexin V-FITC-positive/PI negative cells were regarded as a measure of early apoptosis. The Annexin V-FITC-positive/PI positive cells were regarded as a measure of late apoptosis. The Annexin V-FITC-negative/PI-positive cells were considered to include necrotic cells. The percentage distributions of normal, early apoptotic, late apoptotic, and necrotic cells were calculated using FACSDiva software (Becton Dickenson, San Jose, CA).

### TUNEL Assay

Apoptosis was confirmed by Terminal deoxynucleotidyl transferase dUTP nick end labeling (TUNEL) analysis using an *in situ* cell death detection kit (Roche, Mannheim Germany). Cells were seeded (30,000 cells) on chamber slides (Nunc, Denmark) and treated with 300 μg/ml IV extract. After 48 and 72 h, cell morphology was examined using 4′,6-diamidino-2-phenylindole (DAPI) and TUNEL staining. At the end of treatment, cells were washed twice with PBS, fixed for 60 min with 4% paraformaldehyde and then permeabilized, using 0.1% Triton X-100 in 0.1% sodium citrate, to allow penetration of the TUNEL reaction reagents into the cell nucleus. TUNEL reaction mixture (TdT and fluorescein-dUTP) was added to label the fragmented DNA at 37°C for 1 h in humidified atmosphere in dark. After incubation time, cells were washed twice in PBS, and stained with DAPI solution in order to assess total cell number and for visualization of DNA morphology. Finally, the labeled DNA and the nucleus area were visualized by fluorescence microscopy (Nikon, Kawasaki, Japan).

### Western Blot Analysis

Western blot analysis was performed for the assessment of Caspase-3, Caspase-8, Caspase-9, and PARP levels following treatment with 300 μg/ml of IV extract for 14, 24, 48, or 72 h. Cellular lysates were prepared by suspending 1 × 10^6^ cells in glycerol lysis buffer (50 mM HEPES, 250 mM Nacl, 0.5% NP-40, 2 mM EDTA, 10% Glycerol) containing protease inhibitor cocktail (Roche, Mannheim, Germany). The lysates were centrifuged and the supernatants were collected. The protein concentrations were quantified using Bio-Rad protein assay based on the method of Bradford (14). Protein samples (60 μg) were separated on 12% SDS-polyacrylamide gels and electro-transferred to a 0.45 microns pore size nitrocellulose membrane, using semi dry transfer. The membrane was blocked in 5% non-fat dry milk in Tris-buffered saline and 0.1% Tween 20 (TBST) buffer and incubated with appropriate monoclonal primary antibodies: Anti-caspase 3; 1:5,000, Anti-caspase 8; 1:1,000 (Abcam, Cambridge, UK), or polyclonal primary antibodies: human specific Anti-caspase 9; 1:1,000, PARP antibody; 1:1,000 (Cell Signaling Technology, MA, USA), in a blocking buffer overnight at 4°C. After primary antibody incubation, the membrane was washed three times in TBST and incubated with appropriate secondary horseradish peroxidase-conjugated antibody (Jakson Immuno Research, USA) for 1 h at room temperature, followed by three washes with TBST. The membrane was developed using the EZ-ECL Chemiluminescence detection kit for HRP (Biological industries, Kibbutz Beit Haemek, Israel) and signals were observed and documented using gel imager ChemiDOcTMXRS Gel Documentation System (Bio-Rad, CA, USA). β-Actin (MP Biomedicals OH, USA) was detected on the same membrane and used as a loading control. Quantification of β-Actin normalized immunoblotting was carried out by densitometry using TotalLab TL120 graphic software (Non-linear Dynamycs LTD, NC, USA).

### *In vivo* Studies

#### Animals

The therapeutic activity of *Inula viscosa* extract was investigated using 6 week-old (20–25 g body weight) male C57BL/6 mice (Harlan Laboratories, Jerusalem, Israel). Mice were maintained in a standard cage (5 mice per cage) under sterile condition; with air filter tops and in a filtered laminar air flow room, temperature of 22°C, and under 12 h light/dark schedule at the animal house at the Technion-Israel Institute of Technology (Haifa, Israel). Rodent diet (Koffolk Inc., Tel-Aviv, Israel) and tap water were autoclaved and provided *ad libitum*. The mice were kept in the animal facility at least 1 week before starting the experiments.

### *In vivo* Tumor Growth Assay

MC38 cells (10^6^) suspended in DMEM medium (0.2 ml) were injected subcutaneously (SC) into the right dorsal flank of the mice, using 25-gauge needle (Terumo scientific Inc.). When the tumor size reached about 100 mm^3^ (about 2 weeks after cell implantation), the animals were divided into 4 groups (*n* = 8) (2 control and 2 treated groups). One group served as a control group that was not transplanted with M38 cells (control 1). Three groups were transplanted with M38 cells, while two groups were treated with 150 or 300 mg/kg, and the third group was treated with PBS (control 2) by intraperitoneal (IP) injection. Treatments were conducted three times a week for 3-weeks and body weights and tumor volumes were measured biweekly. Body weights were measured using a weighing scale (Precisa, Switzerland) and tumor volumes were measured with digital caliper and calculated using the formula: length × width^2^ × 0.52, where width represents the shortest dimension of the tumor (15). At the end of treatment period, two mice from each group were taken to ultrasound imaging, and finally mice were sacrificed, tumors were surgically excised and their final weights and volumes were measured and tested for histological studies.

Blood samples were also collected from mice for analysis of liver and kidney functions.

### Ultrasound Imaging

For ultrasound imaging, the VEVO 2100 high frequency ultrasound system (VisualSonic, Inc, Toronto, Canada) was used. Mice were anesthetized with 1.5% isoflurane in oxygen delivered via nose cone and allowed to breathe spontaneously. Mice were positioned on a feedback-controlled heating MousePad. The hair was shaved, in the area overlaying the tumor, with hair removal cream and pre-warmed ultrasound gel (ECO-MED Diagnostic Imaging, Canada) was applied on the skin of mice. Tumors were circled and measured using software incorporated in to the VEVO 2100 device.

### *In situ* Cell Analysis

When the mice were sacrificed, tumors were excised and kept in formalin, and then paraffin blocks were prepared. Four micron sections were cut and fixed onto slides for Ki-67 staining and TUNEL assay.

### Ki-67 Staining

Ki-67 is as large nuclear protein, preferentially expressed during all active parts of the cell cycle (G1, S, and G2/M), but absent from resting cells (G0). Cell proliferation in the tumors was analyzed by immunohistochemistry with formalin fixed section stained with anti-rat Ki-67 antigen antibody (DakoCytomation, Glostrup, Denmark), according to manufacturer instructions. Pre-treatment of tissue sections was performed using PT system (Dako, Glostrup, Denmark) and EnVision™FLEX, High pH (Link) kit (Dako, Glostrup, Denmark). Briefly, Target Retrieval Solution (provided with the kit) was used at 97°C for 20 min and then at 60°C for more 20 min. Slides were treated with peroxidase block (Envision FLEX Peroxidase-Blocking reagent, provided with the kit) for 5 min, and rinsed with buffer for 5 min. Primary rat Ki-67 antibody was applied at 1:50 dilution for 30 min at room temperature. Then, slides were rinsed twice for 5 min and secondary antibody (EmVision FLEX/HRP, provided with the kit) was applied at 1:300 dilutions. Slides were washed three times for 5 min in buffer and visualized using DAB+ (provided with the kit) as chromagen and counterstained with hematoxylin.

### TUNEL Assay and DAPI Staining

Paraffin-embedded sections of tumors were fixed in 4% paraformaldehyde and dewaxed. Briefly, slides were deparaffinized by heating at 60°C for 1 h in a hybridization oven. Next, slides were placed in a plastic slides holder and filled with W-CAP citrate buffer pH = 6 (Bio-Optica, Milan, Italy). The slides holder was placed in a water bath set to 65°C with shaking for 20 min. Slides were washed twice with DDW for 5 min and stripped from proteins by incubation with 20 μg/ml proteinase K (PK) (Sigma-Aldrich Israel, Rehovot, Israel) for 15 min at room temperature, and then washed in DDW for 2 min. TUNEL assay (Roche, Mannheim Germany) was performed according to the instructions by the manufacturer and stained with DAPI solution. At the end, slides were visualized by fluorescence microscopy.

### Statistical Analysis

All experiments, except the *in vivo* studies, were repeated at least three times (unless indicated otherwise). All data were expressed as mean value ± Standard Error (SE), and the statistical differences between groups were evaluated using Student's *t*-test for comparison between two groups or ANOVA test (or their non-parametric counterparts) for comparison between multiple groups. *P* < 0.05 was considered statistically significant and the SPSS software was used for the calculation of differences.

## Results

### *In vitro* Studies

#### Inhibition of Colon Cancer Cell Growth by Inula Viscosa Extract

**Figure 2:**
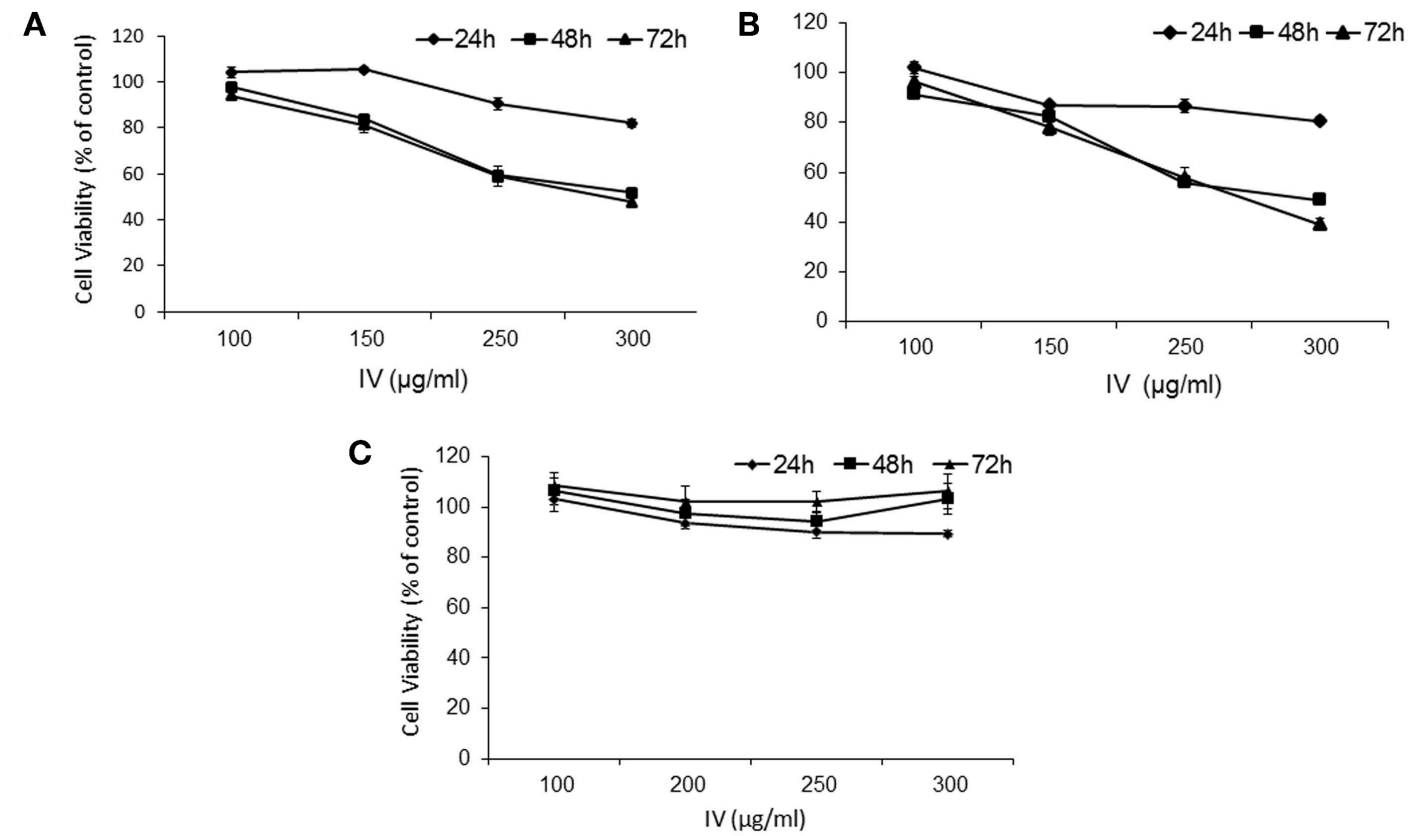
IV treatment reduces cell viability of human colorectal cancer cells. HCT116 **(A)**, Colo320 **(B)** cells, and normal primary liver fibroblasts **(C)** were treated with increasing concentrations of IV extract (100–300 μg/ml) for 24, 48, and 72 h and cell viability was determined using XTT assay as described under “Materials and Methods.” The results are presented as the percentage of control and expressed as mean ± SE of four independent experiments in which each treatment was performed in five replicates.

The results indicated that water extract of IV significantly (*P* < 0.001) decreased cell viability of HCT116 (Figure 2A) and Colo320 (Figure 2B) cells in a time and dose dependent manner. By contrast, cell viability of normal primary liver fibroblasts showed no significant change following treatment with IV extract, in comparison to untreated cells (Figure 2C). Cells were cultured in the presence of escalating concentrations of IV extract (100–300 μg/ml) on 96-wells for different times of treatment (24–72 h), and cell viability was measured by XTT assay (Figure 2). Cell viability, as measured for HCT116 (Figure 2A) was decreased by 18 ± 1.88%, 48.2 ± 7.77%, and 52.3 ± 3.81% (*p* < 0.001) following 24, 48, and 72 h of treatment with 300 μg/ml of IV extract, respectively. Cell viability of Colo320 (Figure 2B) decreased by 19.35 ± 1.5%, 51.2 ± 1.97%, and 60.89 ± 2.36% (*p* < 0.001) following 24, 48, and 72 h of treatment with 300 μg/ml of IV extract, respectively. The IC_50_ values were determined and found to be ~300 μg/ml for both cell lines. For further *in vitro* experiments, the concentration of 300 μg/ml was chosen.

In order to exclude the possibility of cytotoxic effects of IV extract on the cells, Lactate dehydrogenase (LDH) leakage assay was performed. The assay detects the release of stable cytosolic enzyme LDH into the culture medium, due to cell membrane damage, as a result of cell lysis or injury, regardless to the type of cell death (16). Cells were treated with increasing doses (50–350 μg/ml) of IV extract for 24 h. It was observed that IV extract, at concentration of up to 350 μg/ml does not cause a statistically significant change in LDH level in the media, compared with untreated control cells (Data not shown).

### The Effect of *Inula viscosa* on Cell Cycle Progression

**Figure 3:**
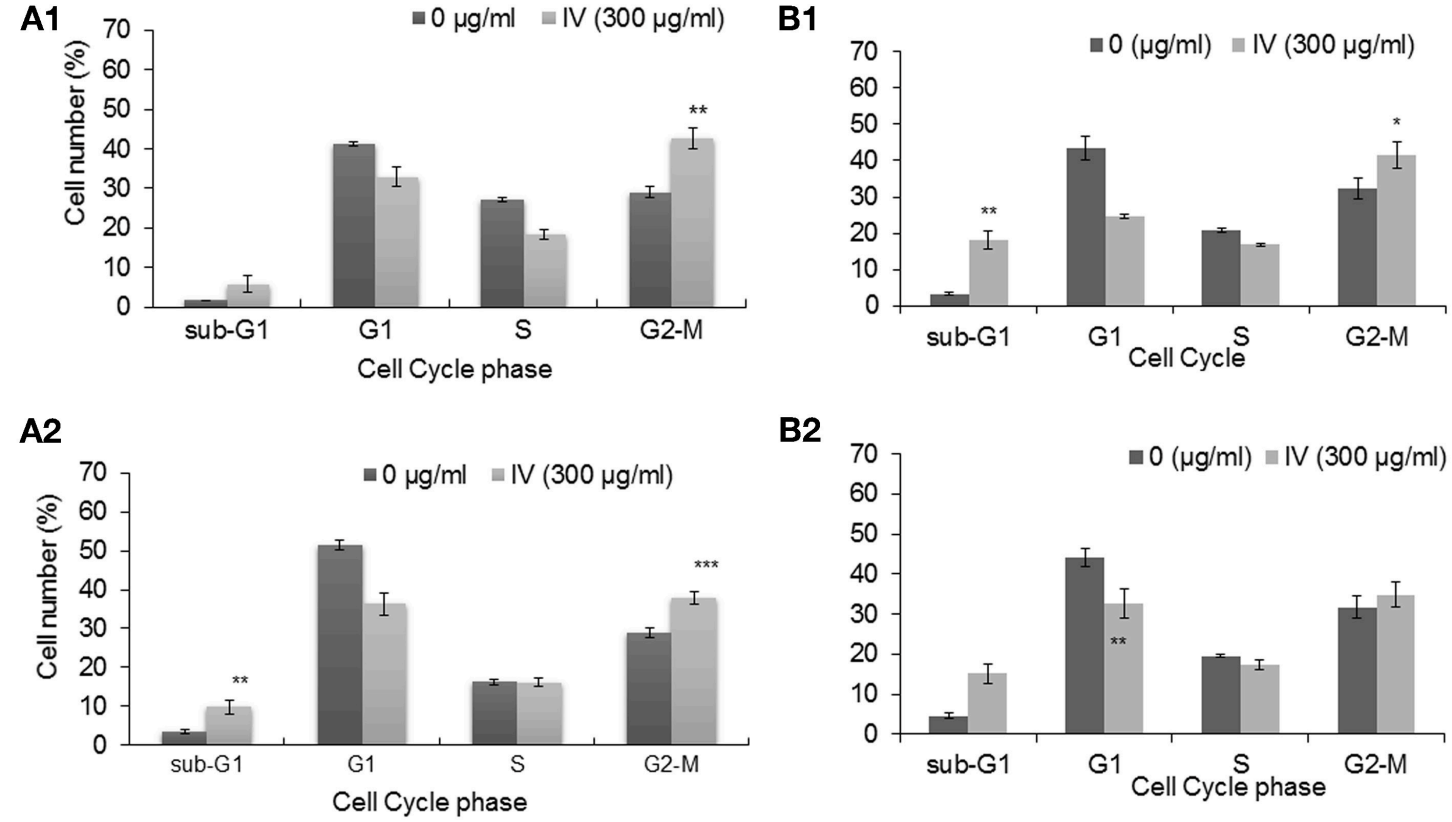
Treatment with IV for 14–24 h resulted in cell cycle arrest at G2/M phase. HCT116 **(A)** and Colo320 **(B)** cells were treated with 300 μg/ml of IV extract for 14 h **(A1,B1)** and for 24 h **(A2,B2)** and subjected to cell cycle analysis by flow cytometry. The results are presented as mean ± SE of five experiments each conducted in duplicates and are expressed as percentage from total 10,000 analyzed cells. Statistical significance was determined by a two-tailed student's *t*-test (treatment VS. control) and is indicated as ^*^p < 0.05; ^**^p < 0.01; ^***^p < 0.001.

**Figure 4:**
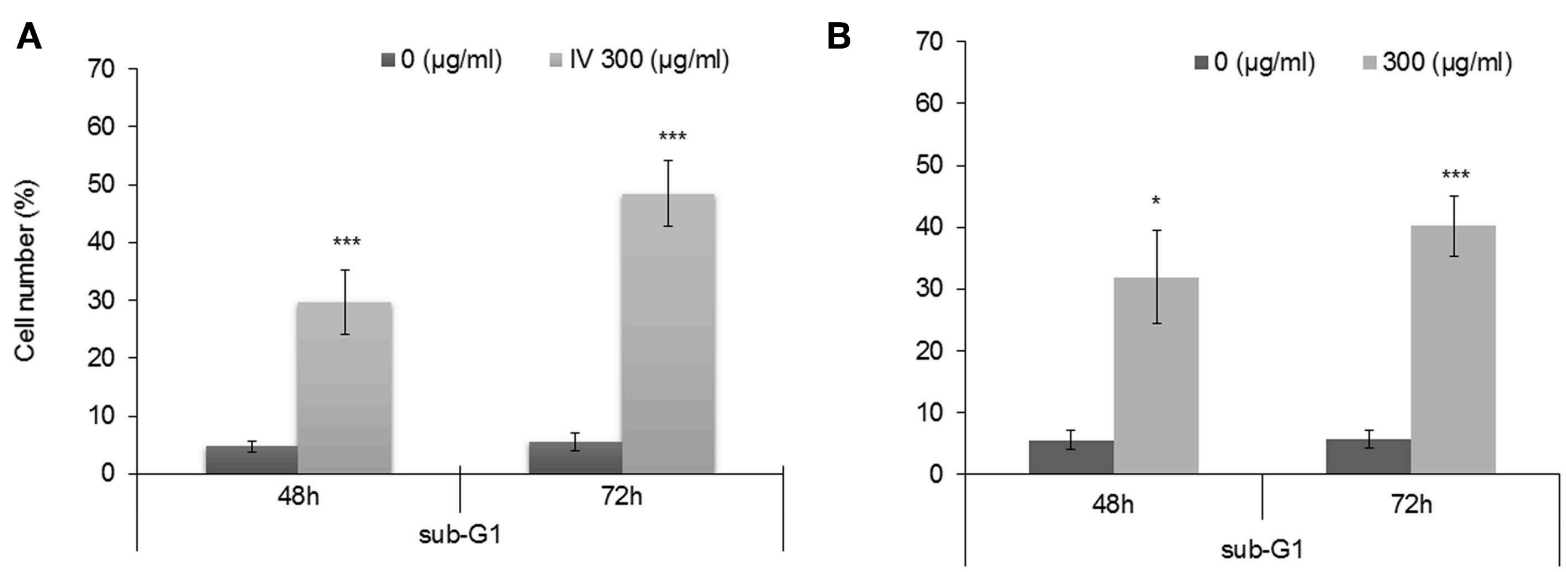
Treatment with IV for 48–72 h resulted in accumulation of cells in Sub G1 phase of the cell cycle. HCT116 **(A)** and Colo320 **(B)** cells were treated with 300 μg/ml of IV extract for 48 h and for 72 h and subjected to cell cycle analysis by flow cytometry. Data are presented as mean ± SE of five experiments, each conducted in duplicates and are expressed as percentage from total 10,000 analyzed cells. Statistical significance was determined by a two-tailed student's *t*-test (treatment vs. control) and is indicated as ^*^p < 0.05; ^***^p < 0.001.

In order to study the effect of IV extract on cell cycle progression, cells were incubated with 300 μg/ml of IV for 14–72 h (Figures 3, 4), and the distribution of cells in the different phases of cell cycle was determined by fluorescence activated cell sorter (FACS). The results indicated that exposure of HCT116 and Colo320 to 300 μg/ml of IV extract exerted inhibitory effects on the cell cycle progression in a time dependent manner. Exposure of HCT116 cells to IV extract resulted in an increased proportion of cells in the G2/M phase of the cell cycle after 14 h (*P* < 0.01; Figure 3A1) and 24 h (*P* < 0.001; Figure 3A2) of treatment, as compared with untreated cells (control). Similarly, exposure of Colo320 cells to 300 μg/ml of IV extract resulted in an increased proportion of cells in G2/M phase following 14 h (*P* < 0.05; Figure 3B1) and no significant increase after 24 h (Figure 3B2) of treatment, as compared with the untreated cells. Extended periods of treatment (48 and 72 h) caused cells to exit from G2/M phase toward the sub-G1 phase (Figure 4), indicating the possibility of induction of apoptosis (17). A significant increase of cell accumulation in sub-G1 phase compared to untreated cells, has been observed following 48 and 72 h of treatment, in both cell lines (HCT116, 48.47 ± 5.67% vs. 5.54 ± 1.52% after 72 h; Colo320, 40.18 ± 4.81% vs. 5.68 ± 1.48%, after 72 h, Figures 4A,B, respectively). This may indicate that exposure of the cells to IV extract for 48–72 h induces programed cell death.

### Treatment With IV Extract Induced Apoptosis in Colorectal Cancer Cells

**Figure 5:**
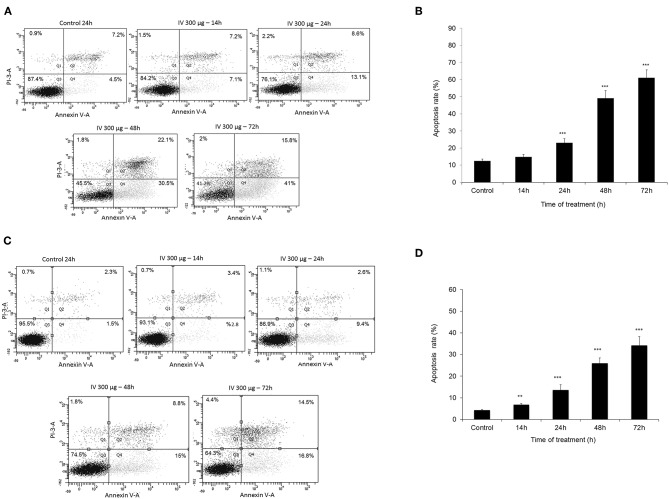
Measurement of apoptotic cells using an Annexin V binding. HCT116 **(A,B)** and Colo320 **(C,D)** cells were treated with 300 μg/ml IV for 14–72 h. and Flow cytometric analysis of Annexin V-FITC/PI double-stained was performed. In each plot **(A,C)** the lower left quadrant (Q3) represents viable cells, the upper left quadrant (Q1) indicates necrotic cells, the lower right quadrant (Q4) denotes early apoptotic cells, and the upper right quadrant (Q2) represents necrotic or late apoptotic cells. Data are presented as mean ± SE of five independent experiments, each conducted in duplicates [mean (Q2+Q4) ± SE] **(B,D)**. Statistical significance was determined by a two-tailed student's *t*-test [treatment vs. control (untreated cells)] and is indicated as ^**^p < 0.01; ^***^p < 0.001.

**Figure 6:**
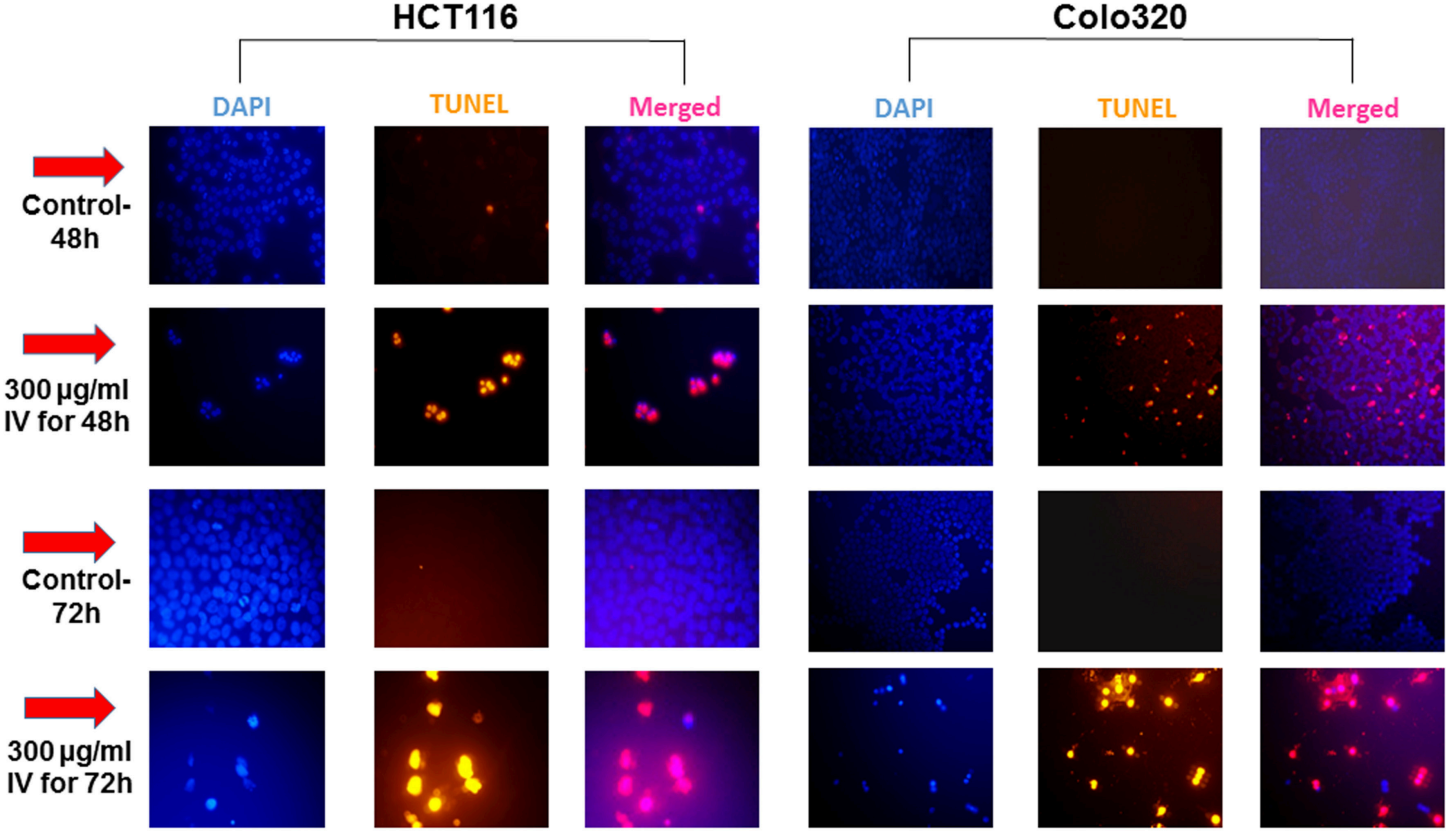
IV treatment of colorectal cancer cells induced apoptosis. HCT116 and Colo320 cells were seeded in chamber slides and treated with 300 μg/ml IV extract for 48 or 72 h. The cells were analyzed for apoptosis by TUNEL assay. Illustrations are representatives of three independent experiments, each conducted in duplicate. Blue-colored cells (DAPI dye) are living cells; red colored cells (fluorescence-labeled dUTP) are apoptotic cells. Cells were visualized by fluorescence microscopy (original magnification X20).

Annexin V-FITC and propodeum iodide (PI) staining were used in order to quantify the percentage of cells undergoing apoptosis, necrosis and viable cells. The apoptotic cells were counted as early apoptotic cells (quadrants Q2), late apoptotic cells (quadrants Q4), and represented as percentage of apoptotic cells (Figure 5) from total cell population. Figures 5A,B, indicate that treatment of HCT116 cells with 300 μg/ml of IV extract for 24, 48, or 72 h resulted in a significant increase of Annexin V by 2, 4.2, and 5.1-folds, respectively. Similarly, treatment of Colo320 cells with 300 μg/ml of IV extract for 14, 24, 48, or 72 h significantly increased Annexin V by 1.6, 3.16, 6.04, and 7.95-folds, respectively, compared to control (Figures 5C,D). These results indicated that IV induces apoptosis on colorectal cancer cells. Terminal deoxynucleotidyl transferase dUTP nick end labeling (TUNEL) assay confirmed that exposure of the cells to IV extract induces apoptosis (Figure 6). HCT116 and Colo320 cells were grown in the absence or presence of 300 μg/ml of IV extract for 48–72 h. As shown in Figure 6, extensive DNA fragmentation was visible by fluorescence microscopy after TUNEL staining following treatment, compared to untreated cells.

### *Inula viscosa* Extract Activates Caspases

**Figure 7:**
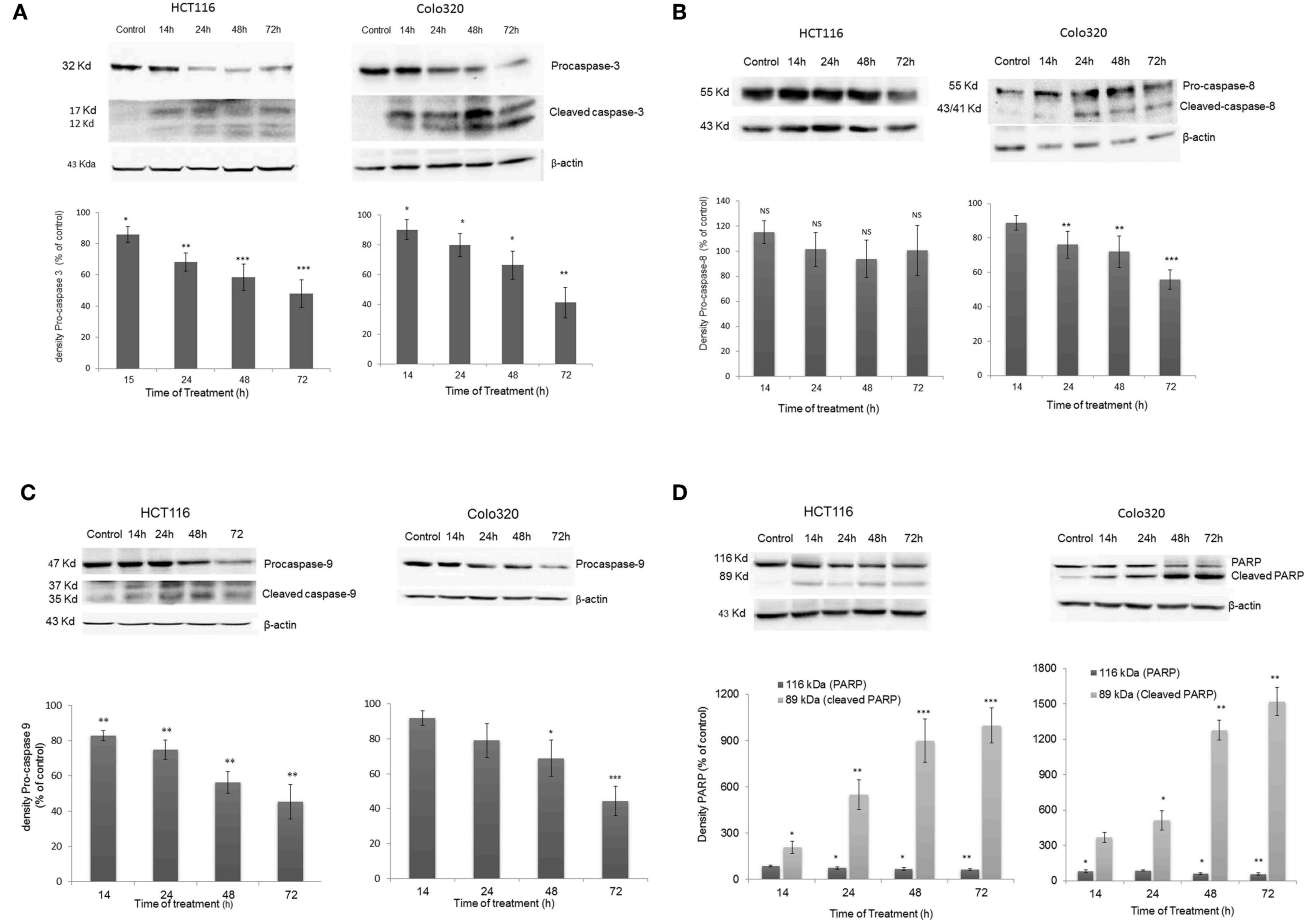
Activation of Caspases following IV treatment. HCT116 and Colo320 cells were treated with 300 μg/ml IV extract for 14–72 h. and caspase−3 **(A)**, caspase−8 **(B)**, caspase−9 **(C)**, and PARP **(D)** were detected by Western blot, where β-Actin was used as loading control. Densitometry analysis represents the average expression levels of the pro-caspase and/or the cleaved caspase and PARP; Density values were calculated as a control from the proper β-actin and as a percent of control. Data represented are average of three independent experiments (mean ± SE). Statistical significance was determined by a two-tailed student's *t*-test (treatment vs. control) and marked as ^*^p < 0.05, ^**^p < 0.01, ^***^p < 0.001; NS, Not Significance.

To further investigate the mechanism of action of cell death induced by IV extract, a western blot analysis was performed to detect proteins that have been shown to be involved in both the extrinsic (caspase-8) and the intrinsic (caspase-9) apoptosis pathways. Data presented in Figure 7, showed that treatment of HCT116 cells with IV extract for 14–72 h activated caspase-9 (Figure 7C), but not caspase 8 (Figure 7B), followed by activation of caspase-3 (Figure 7A) and cleavage of Poly (ADP-ribose) polymerase (PARP) (Figure 7D). This may suggest that IV induced apoptosis in HCT116 cells through the intrinsic pathway. Moreover, data presented in Figure 7 showed that treatment of Colo320 cells with IV extract for 14–72 h clearly activated caspase-8 (Figure 7B). In Figure 7C a gradual decrease of pro-caspase-9 was seen, but not a clear cleavage. The activation of caspase-3 was well observed (Figure 7A) as well as the cleavage of PARP (Figure 7D). This may suggest that IV induced apoptosis in Colo320 cells through the extrinsic pathway and probably via the intrinsic pathway as well.

### *In vivo* Studies

The effect of IV leaves water extract on tumor growth was examined on subcutaneously transplanted mouse tumor cell line, MC38, in C57BL/6 mice. Prior to preforming the *in vivo* experiments, the effects of IV on cell viability, cell proliferation, cytotoxic effects, and apoptosis induction were examined *in vitro* on MC38 cell line. The effects of IV extract on MC38 cells were similar to the results obtained with HCT116 and Colo320 cells.

### *Inula viscosa* Extract Inhibits Tumor Growth in Animals

**Figure 8:**
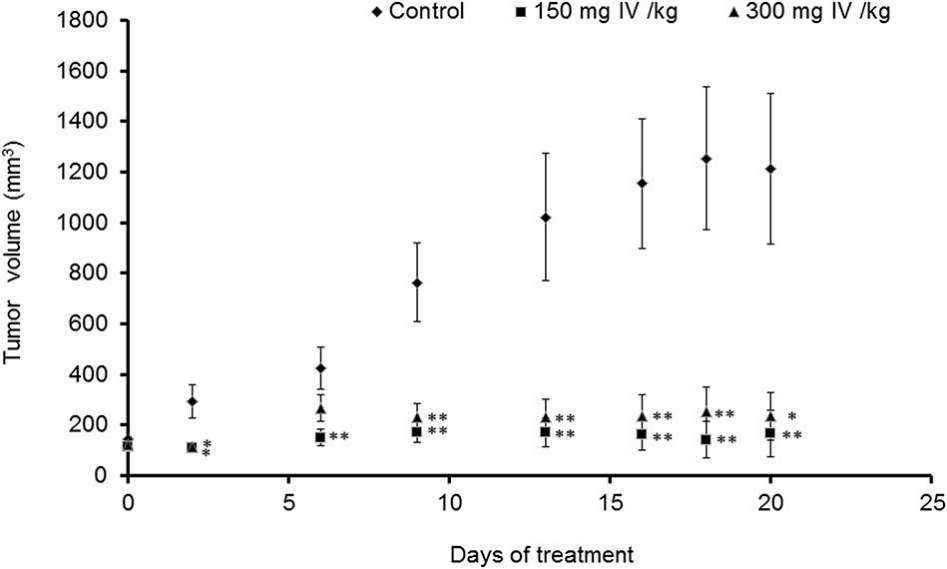
IV extract inhibited tumor growth of colon cancer cells in mice. C57BL/C male mice were subcutaneously (SC) implanted with 1 × 10^6^ MC38 cells. When the tumors reached a volume of 100 mm^3^, mice were treated 3 times a week by intraperitoneal injection (IP) of either PBS × 1 to the control group, or IV extract, (150 or 300 mg/kg body weight) for 3 weeks, as described under “Materials and Methods.” During the experiments, tumor volumes were measured twice a week using caliber meter. The results are presented as the mean ± SE. *n* = 8 mice per experimental group. Statistical significance was determined by a two-tailed student's *t*-test (treatment vs. control in each time point) and assigned as ^*^p < 0.05, ^**^p < 0.01 and by Repeated Measures ANOVA with Tukey HSD *post hoc* test for the evaluation of changes in tumor volumes over time in each group and for differences between control and treatment groups.

**Figure 9:**
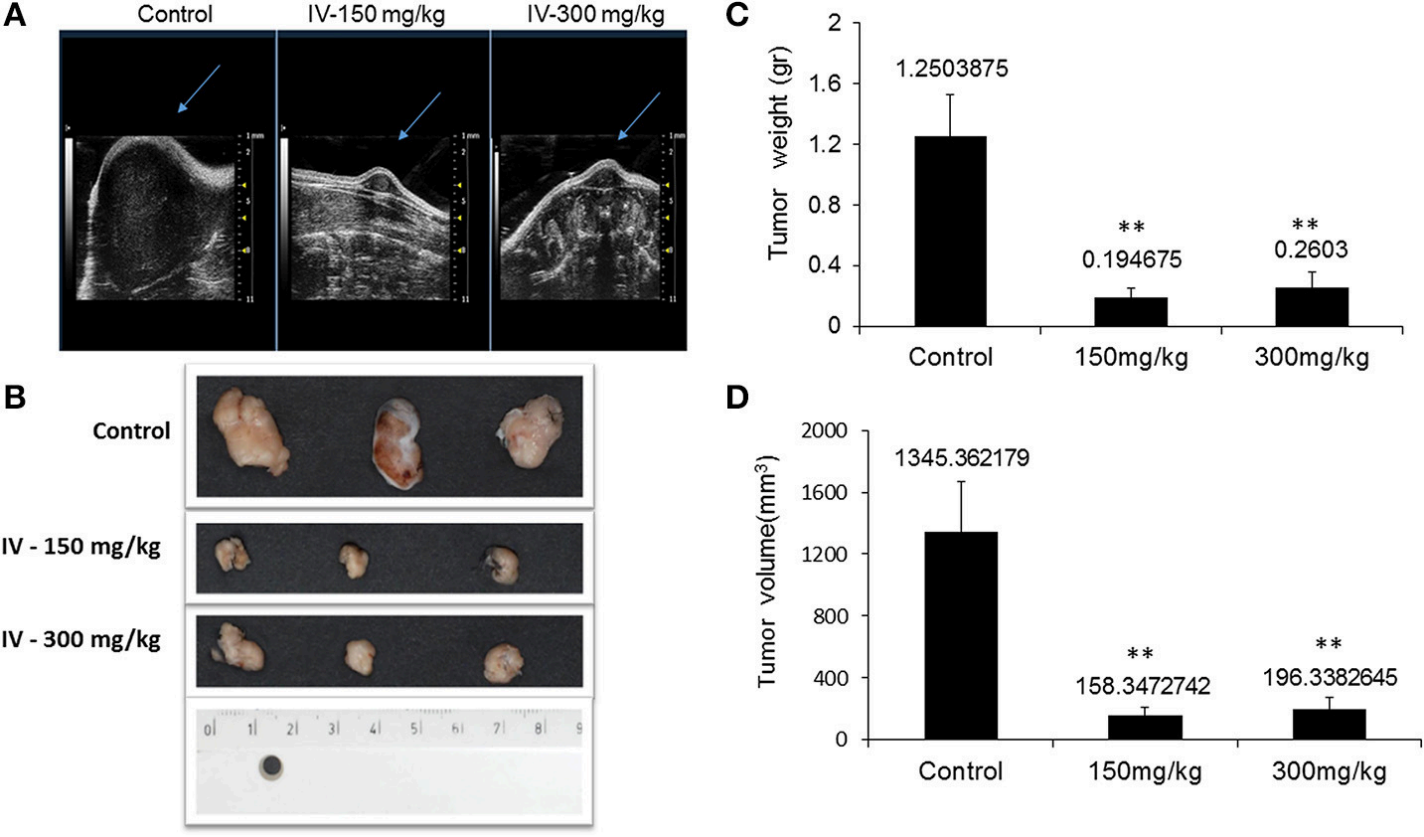
IV aqueous extract suppressed tumor growth. Mice were anesthetized with 1.5% isoflurane in oxygen delivered via nose cone and allowed to breathe spontaneously. Ultrasound imaging was performed using the VEVO 2100 high frequency ultrasound system **(A)**. At the end of the treatment period, mice were sacrificed, tumors were isolated and pictures of the tumors from control or treated (150 or 300 mg IV extract per kg) groups were taken **(B)** and the final tumor weights **(C)** and volumes **(D)** were measured. The results are presented as the mean ± SE of *n* = 8 tumors per experimental group. Statistical significance for tumor weight was determined by Mann-Whitney and for tumor volume by two-tailed Student's *t*-test (treatment vs. control). ^**^p < 0.01.

Animals were injected with MC38 cells subcutaneously into the flank. Two weeks after tumor formation, animals were treated intra-peritoneum (IP) with either PBS or IV extract (150 and 300 mg/kg) three times a week for 3 weeks. Tumor volumes were measured twice a week. The results indicated that treatment with IV extract significantly (*P* < 0.001) decreased tumor growth in comparison to control group (Figure 8). While the tumor volumes in the control group increased by ~8.5-folds over the treatment period, the tumor volumes of the treated group with 150–300 μg/kg IV were decreased by 1.9 and 1.2-folds, respectively. At the end of the treatment period (day 21), ultrasound imaging indicated that treatment with IV resulted in a reduction *in situ* of the tumors compared to control (Figure 9A). Figure 9B demonstrates representative samples of the tumors collected after scarifying the animals and dissection of the tumors. As can be seen, there is an enormous difference between the tumors from control and treated mice.

The results also indicated that the tumor weights (Figure 9C) and tumor volume (Figure 9D) were significantly (*P* < 0.001) decreased.

In order to reveal any cytotoxic effect of IV extract *in vivo*, the average body weights of untreated and treated mice were measured every alternate day. The body weight did not vary significantly throughout the study, according to Repeated Measures ANOVA test; [*F*_(6.744, 62.944)_ = 0.727, *P* = 0.645] (data not shown). Moreover, there were no observable signs of distress such as impaired movement, ruffled fur or change in behavior of IV-treated animals compared to controls. In addition, kidney and liver functions were measured in blood samples collected from control and treated animals at the end of the experiment following animal sacrificing. The results indicated that there was no significant difference in liver and kidney parameters that were measured in control and treated animals (Table 1).

**Table 1:** IV extract did not affect kidney and liver functions.

**Serum chemistry**	**Control 1**	**Control 2**	**IV 150 mg/kg**	**IV 300 mg/kg**	***p*-value (1)**	***p*-value (2)**
**KIDNEY FUNCTION**						
UREL (mg/dL)	47 ± 3.78	55.6 ± 5.87	51.5 ± 2.13	48.7 ± 2.61	0.43	0.231
NA-I (mmol/L)	156.96 ± 8.95	152.32 ± 0.52	151.48 ± 1.39	151.23 ± 1.55	0.687	0.638
CA (mg/dL)	8.74 ± 0.55	11.15 ± 0.28	10.80 ± 0.19	10.5 ± 0.28	0.317	0.175
K-I (mmol/L)	14.64 ± 17.69	7.39 ± 0.62	9.09 ± 0.75	8.80 ± 0.69	0.167	0.217
Creatinine (mg/dL)	< 0.2	< 0.2	< 0.2	< 0.2		
**LIVER FUNCTION**						
ALT (u/l)	59.78 ± 9.39	99.28 ± 41.39	114.82 ± 63.53	67.48 ± 12.81	0.756	0.569
ALP2L (U/L)	64 ± 8.84	87 ± 3.86	85 ± 10.86	77.11 ± 6.29	0.865	0.206
AST (U/L)	323.94 ± 71.4	244.16 ± 64.4	269.30 ± 68.8	380.15 ± 87.3	0.803	0.309
GGTI (U/L)	6.9 ± 1.68	0.32 ± 0.25	1.28 ± 0.67	3.37 ± 2.92	0.331	0.486

*The effect of IV on kidney and liver functions following animal treatment with 150 mg/kg and 300 mg/kg for 3 weeks was tested. Control 1 represent animals without tumors and control 2 represent animals with transplanted tumors. Control groups were treated with saline. Data are expressed as mean ± SE of 8 animals in each group. p-values are calculated when comparing IV treated 150 mg/kg [p-value (1)] or 300 mg/kg [p- value (2)] with untreated mice. Statistical significance was determined by two-tailed student's t-test or Mann-Whitney*.

### *Inula viscosa* Extract Inhibits Cell Proliferation and Induced Apoptosis of Colorectal Cancer Tumors in Mouse Model

**Figure 10:**
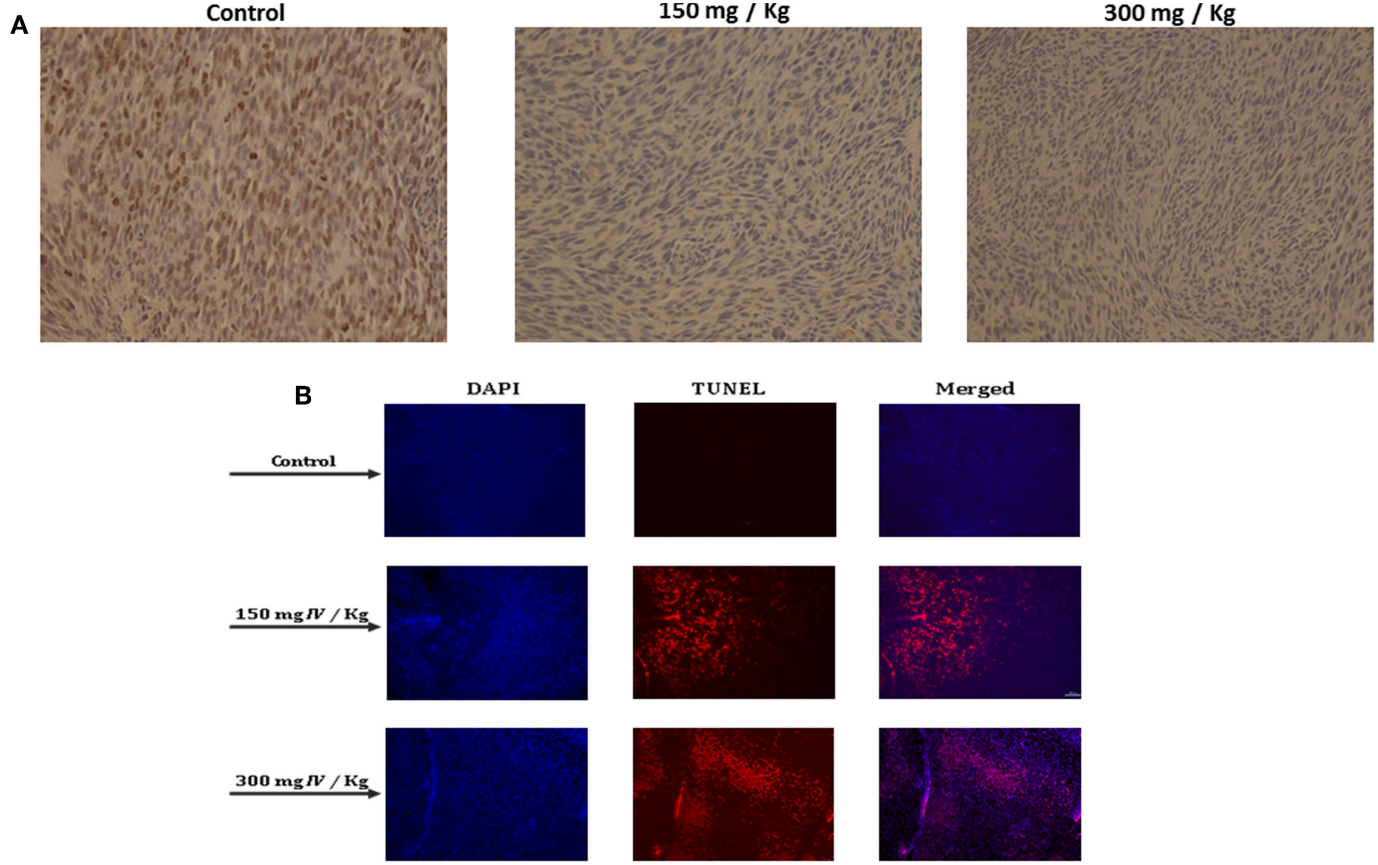
IV inhibited cell proliferation and induced apoptosis in transplanted tumors in mice. Paraffin-embedded tissue sections were prepared from the tumor tissues and the **(A)** proliferation profiles of the cells were measured by detection of Ki-67. The images are representatives of the results obtained from control and treatment groups (Magnification, 200X). **(B)** DAPI and TUNEL staining of tumor sections. Tumors tissues were stained with DAPI and TUNEL and analyzed under a fluorescent microscopy. The TUNEL positive (shining orange) cells are apoptotic cells, nuclei are labeled with DAPI (blue) and the merge between DAPI and TUNEL appears pink (Magnification, 200X).

In order to better understand how IV extract affects tumor growth *in vivo*, tumors were collected, fixed in formalin, and sections were prepared for several histological staining. In order to examine the effect of IV extract on cell proliferation, cells were stained with Ki-67 antibody. The results indicated that the number of proliferating cells in the control specimens is larger than that seen in the specimens of treated animals (Figure 10A). This may indicate that IV treatment reduced tumor cell proliferation. In addition, the tumor specimens were stained with DAPI and TUNEL (Figure 10B). The results revealed that treatment with IV extract resulted in a morphological changes and induction of apoptosis.

These results strongly demonstrated that treatment with IV aqueous extract reduced cell proliferation and induced apoptosis of colon cancer cells that resulted in the inhibition of tumor growth in the allograft mice model.

## Discussion

The treatment of cancer over the past decades has relied primarily on the use of various forms of cytotoxic chemotherapy and radiation therapy. The effectiveness of cytotoxic treatments has been limited by the side effects and by the development of resistance. In order to combat the problem of side effects, selectivity, and drug resistance, new effective drugs are needed for CRC treatment. Natural products are a source of anticancer drugs which frequently seem to be more effective and/or less toxic (6). Approximately 60% of drugs that are currently in use for cancer treatment, have been isolated from natural products and many of them are derived from plants (18). In many countries around the world, medicinal plant extracts constitute a common alternative for cancer treatment, with no toxicity (19).

The present study was undertaken to evaluate the anticancer effects of *Inula viscosa* (IV) leaf water extract, on the growth of human colorectal cancer cells *in vitro*, and on tumor growth *in vivo* using a xenograft mouse model.

The extract showed profound anti-cancer effects on two human colorectal cancer cell lines; HCT116 and Colo320. Cell viability was significantly reduced following exposure to IV extract in a time and dose dependent manner with IC_50_ of ~300 μg/ml without affecting LDH release.

Different solvents used for IV extractions revealed different molecules present in the extract with dependency to the solvents used. For example, a recent study revealed the presence of 51 compounds, including 11 phenolic acids, 23 flavonoids, one lignin, and 12 terpenoids in ethanol extract of IV leaves. Twenty six of these compounds were described for the first time in IV (20). Several studies have shown the anti-proliferative activity of IV extracted from different parts of the plant and by different solvents. Previous works reported that *in vitro* proliferation of breast cancer cells, MCF-7, was decreased after exposure to flower IV extracts, derived by different solvents (11). Other studies, have demonstrated the cytotoxic effect of hexanic extract of IV leaves on human cervical carcinoma cell lines, *in vitro* (21, 22).

In the present study, we report the anti-proliferative activity of water extract from IV leaves on human colorectal cancer cells. *Inula viscosa* leaves are known to contain many biological active compounds (23), including polyphenols such as phenolic acids that seem to be the most abundent group of polyphenols in IV leaves (24). Some of the polyphenol classes found in IV leaves are flavonoids, terpens, lactones (9, 10, 25, 26), and sesquiterpens acids such as Tomentosin and Inuvisocolide (12). Polyphenol fraction from IV leaves was recently investigated, by Brahmi-Chendoyh et al. (27) and was found to be rich in caffeoyl shikimic acids and unusual dihydrobenzofuran ligands as main secondary metabolites. Moreover, 43 secondary metabolites were identified in this fraction (27). The process of carcinogenesis might be stopped or delayed by exposure to phytochemicals via diet or pharmacological administration (28). For instance, phytochemicals are thought to potentially have a role in reducing the incidence of colon cancer (29). The Polyphenols, including flavonoids and phenolic acid, and sesquiterpens, are noted for their ability to induce apoptosis in colon cancer cells (30, 31). Moreover, high levels of reactive oxygen species (ROS) cause several disorders including progression of multiple cancers, including colorectal cancer (32). It is well known that the main role of antioxidants is to protect against damage caused by ROS, leading to a reduction of cancer risk (32, 33). Extract from IV leaves was found to have antioxidant activity as well as polyphenols, such as 1,3-dicaffeoylquinic acid and more different derivatives of dicaffeoylquinic acid and caffeoylquinic acid. (9, 27). It is well known that the main role of antioxidants found in nutraceuticals is to protect the cells from damage caused by reactive oxygen species (ROS) and reducing cancer risk (32, 33). In light of these findings, it can be assumed that the leaf extract of *Inula viscosa* contains a diversity of active compounds, which exhibit a synergetic or at least, an additive anti-tumor effect on colorectal cancer cells.

The results of the present study indicated that IV extract induces cell cycle arrest at G2/M-phase followed by accumulation of cells in sub-G1 phase. We assume that IV extract induces apoptosis of colorectal cancer cells. Previous studies have demonstrated that there is a link between cell cycle regulation and cancer progression. Therefore, management of cancer can be achieved by targeting and inhibiting the cell cycle progression (34).

The results in the present study are compatible with those of other studies; Belayachi et al. (35) revealed that exposure of PC-3 prostate cancer cells in culture to dichloromethane extract or hexanic extract of *Inula viscosa*, resulted in induction of G2/M arrest, which was detectable at 24 h of treatment and enhanced after 48 h. However, treatment of SW620-colon cancer cells with the same extracts for 48 h caused cell cycle arrest in G1 phase and an increase in the sub-G1 population (35). In addition, Tomentosin and Inuviscolide, two isolated compounds from the *Inula viscosa* leaf extract, induced cell cycle arrest in melanoma cells at the G2/M phase following 4 h of treatment. Prolonging the exposure time to 24 h resulted in a profound peak at the G2/M phase and after extended periods of time, a profound accumulation of cells in sub-G1 phase was observed (12).

Many anticancer drugs act by blocking one or more stages of the cell cycle and finally trigger apoptosis (36). The G2/M phase accumulation is often related to induction of apoptosis in p53 functioning cell lines (37). p53 is a tumor suppressor gene that functions especially as a transcription factor by activating and down regulating gene expression, leading to cell cycle arrest or apoptosis (38). Fischer and colleagues found an involvement of 210 genes regulating G2/M cell cycle, at HCT116 cells, by the discovered p53-p21-DREAM-CDE/CHR pathway. Down regulations of most of these genes, seems to be a major mechanism for the G2/M cell cycle arrest by p53 (38). This may stand also for the G2/M arrest in our experiments in HCT116 cells, but more studies are required to confirm the involvement of p53. Since Colo320 cells are p53 mutant (39), we assume that the activity of IV aqueous extract is exerted in different pathway, which still needs to be explored.

The Annexin-V/PI and TUNEL assays confirmed that the decrease in cell viability was a result of apoptotic processes induced by IV treatment, where the cell cycle arrest was an intermediate stage before apoptotic death (as finally appeared in Sub-G1 ascent). Extension of treatment periods (48–72 h) with IV increases the apoptosis rate in both cell lines. The most obvious characteristics of apoptosis are cytoplasmic and nuclear condensation followed by inter-nucleosomal DNA cleavage, membrane blobbing, and finally cell damage (16). These hallmarks of apoptotic process were observed in the present study.

In most cases, anticancer therapies ultimately result in activation of caspases (40) which play a crucial role in the execution of apoptosis. In the present study, we examined caspase-3 activation at various time points by western blotting analysis, using a polyclonal antibody specific for procaspase-3 and the subunits p12 and p17. According to the results obtained, IV extract induced caspase-3 activation in both cell lines. Caspase-3 was detectable at 14 h after incubation with IV extract and levels of procaspase-3 significantly decreased in a time dependent manner in both cell lines, following treatment.

Subsequently to caspase-3 activation, cleavage of the active form of PARP, which stands for cell survival, is a critical step in the completion of the apoptotic program. Western blot analysis revealed that treatment with IV extract leads to cleavage of PARP in both cell lines in a time dependent manner. The results are correlated with results obtained for caspase-3, in a sense that at all time points at which caspase-3 was activated, PARP was cleaved. These results strengthen the fact that caspase-3 is responsible for the cleavage of PARP during cell death process (41).

In order to reveal whether the apoptotic process occurred through the extrinsic or intrinsic pathway, activation of caspase-8 and caspase-9 was assayed. Interestingly, the results indicated that treatment with IV activated caspase-9 in HCT116, the well differentiated cells, and caspases-8 and probably-9 in colo320 cells, the poorly differentiated cells. These findings may indicate that IV extract induces apoptosis through the intrinsic mitochondrial pathway in well differentiated cells and through both, the intrinsic and extrinsic pathways, in poorly differentiated cells.

The utilization of both extrinsic and intrinsic mechanisms to execute apoptosis can be reasoned by the existing crosstalk between both apoptotic pathways, as described previously by Fulda and Debatin (40). Binding of apoptotic signals causes activation of the extrinsic pathway, including Caspase-8 which activates factors such as Bid. The activated Bid can translocate to the mitochondria and activate the intrinsic pathway, causing initiation of Caspase−9 activity. Activation of Caspases-8 and−9 leads to activation of Caspase-3 which executes the apoptotic process.

Other studies have shown that plant extracts can induce apoptosis through both apoptotic pathways. Lan et al. (42) revealed that a methanolic extract from the *Emilia sonchifolia* plant stimulated the activities of caspases-3,−8, and−9 and promoted the mitochondria-dependent and death receptor-associatedi protein levels in HCT116 cell line (38). Moreover, treatment of HCT116, SW620, and SW480 colorectal cancer cell lines with Grape seed extract resulted in induced cleavage of caspases−3,−8,−9, and PARP. In addition, cytochrome-c was released from the mitochondria of all these three cell lines, suggesting the involvement of both pathways in the apoptotic death (43). To better understand the mechanism of IV induced cell death, additional studies are required.

In the present study, we have demonstrated that IV extract has a potential beneficial effect on the inhibition of tumor growth of subcutaneously transplanted MC38 cells in mice. Treatment with 150–300 mg/kg of IV three times a week for 3 weeks resulted in inhibition of tumor growth compared to controls. Immunohistochemical and histological studies indicated that IV inhibits cell proliferation and induces apoptosis in the tumor cells. On the other hand, treatment with IV extract does not affect body weight and kidney and liver functions. Therefore, it is possible to assume that IV extract is safe and effective in cancer treatment.

To the best of our knowledge, no studies in the literature were found that examine any antitumor effect *in vivo* of any extract from the species of *Inula*. However, numerous studies have evaluated the potential anticancer effects of isolated compounds from the genus *Inula, in vivo*; Bigelovin, a sesquiterpene lactone isolated from *Inula helianthus aquatica*, has been proven to induce apoptosis in CRC through activation of downstream caspase leading to G2/M arrest and DNA damage. Moreover, this compound exhibited potent anti-tumor activities against CRC *in vitro* and *in vivo* (44). Another sesquiterpen, Japonicone A, a dimeric sesquiterpene lactone, isolated from traditional herb *Inula japonica*, has shown potent *in vitro* and *in vivo* anti-tumor activity against Burkitt's lymphoma (45). Flavonoids, a group of polyphenols found in several medical plants, also exhibited antitumor activity *in vivo* in different human cancer cells including CRC cells (46, 47). Interestingly, flavonoids can interact synergistically with other polyphenols in the treatment of cancer to induce apoptosis (30). As mentioned above, *Inula viscosa* leaves contain, among other compounds, sesquiterpens, flavonoids and phenols with proven anticancer activity *in vivo*. These data, together with our *in vivo* results, strengthen the assumption that the different compounds composing the extract probably have a synergistic effect on the pronounced inhibition of tumor growth in mice.

Interestingly, in the present study, no side effects such as weight loss, kidney and liver functions and behavioral changes were observed. It is worth mentioning that, chemotherapeutic agents can produce a variety of acute and chronic organ toxicities including to the liver and kidneys (48, 49). Liver dysfunction under chemotherapy mainly consists of abnormal biologic liver tests indicating chronic cholestasis with elevation in the levels of Alkaline phosphatase (ALP) (49). Under chemotherapy up to 85% of patients develop fatty liver and in the more serious event this condition is accompanied by an increase in bilirubin levels. In addition, repeated chemotherapy induces irreversible hepatocellular damage through recruitment of inflammatory cells (49). More specifically, treatment of patients suffering from metastatic colorectal cancer with oxaliplatin, was strongly correlated to the development of sinusoidal injury in the liver (50). Since many antitumor drugs and their metabolites are cleared via the renal system, the kidneys are exposed to injury (48). Plasma creatinine levels serve as an index of endogenous renal function, with normal values below 0.2 mg/dL in normal mice (51). In the present study, this index did not change compared with control groups (<0.2 mg/dL).

In conclusion, the present study indicated that IV aqueous leaf extract, affects the cell cycle progression and induces apoptosis by activation of caspases in colon cancer cells. Moreover, IV extract exhibits anti-tumor activities in an animal model, and it is safe for use. It can be suggested that IV aqueous leaf extract may serve as a strong potential drug for the treatment and probably for prevention of cancer.

## Ethics Statement

All the experimental animal studies were conducted in accordance with the valid international guidelines after approval by the Animal Ethics Committee at the Technion Institute (Haifa, Israel) (Ethics number IL1481110).

## Author Contributions

FF and RB-S designed the study and wrote the manuscript. RB-S performed the experiments and analyzed the data. MB and SG collected the plants and prepared the extract. NA and LS took part in the *in vivo* studies. All authors reviewed the draft manuscripts, read, and approved the final manuscript.

### Conflict of Interest Statement

The authors declare that the research was conducted in the absence of any commercial or financial relationships that could be construed as a potential conflict of interest.

## References

[1] Ferlay J Colombet M Soerjomataram I Mathers C Parkin DM Piñeros M. Estimating the global cancer incidence and mortality in 2018: GLOBOCAN sources and methods. Int J Cancer. (2018) 144:1941–53. 10.1002/ijc.31937 30350310

[2] Labianca R Beretta GD Kildani B Milesi L Merlin F Mosconi S. Colon cancer. Crit Rev Oncol Hematol. (2010) 74:106–133. 10.1016/j.critrevonc.2010.01.010 20138539

[3] Qureshi A Verma A Ross P Landau D. Colorectal cancer treatment. Clin Evid. (2010) 2010:401. 21718569 PMC2907599

[4] McWhirter D Kitteringham N Jones RP Malik H Park K Palmer D. Chemotherapy induced hepatotoxicity in metastatic colorectal cancer: a review of mechanisms and outcomes. Crit Rev Oncol Hematol. (2013) 88:404–15. 10.1016/j.critrevonc.2013.05.011 23786843

[5] Rabik CA Dolan ME. Molecular mechanisms of resistance and toxicity associated with platinating agents. Cancer Treat Rev. (2007) 33:9–23. 10.1016/j.ctrv.2006.09.006 17084534 PMC1855222

[6] Ma X Wang Z. Anticancer drug discovery in the future: an evolutionary perspective. Drug Discov Today. (2009) 14:1136–42. 10.1016/j.drudis.2009.09.006 19800414

[7] Gordaliza M. Natural products as leads to anticancer drugs. Clin Transl Oncol. (2007) 9:767–76. 10.1007/s12094-007-0138-9 18158980

[8] Wang W Ben-Daniel BH Cohen Y. Control of plant diseases by extracts of *Inula viscosa*. Phytopathology. (2004) 94:1042–7. 10.1094/PHYTO.2004.94.10.1042 18943791

[9] Danino O Gottlieb HE Grossman S Bergman M Antioxidant activity of 1, 3-dicaffeoylquinic acid isolated from *Inula viscosa*. Food Res Int. (2009) 42:1273–80. 10.1016/j.foodres.2009.03.023

[10] Lauro L Rolih C. Observations and research on an extract of *Inula viscosa* Ait. Boll Soc Ital Biol Sper. (1990) 66:829–34. 2073383

[11] Talib WH Mahasneh AM. Antiproliferative activity of plant extracts used against cancer in traditional medicine. Sci Pharm. (2010) 78:33–45. 10.3797/scipharm.0912-11 21179373 PMC3002826

[12] Rozenblat S Grossman S Bergman M Gottlieb H Cohen Y Dovrat S. Induction of G2/M arrest and apoptosis by sesquiterpene lactones in human melanoma cell lines. Biochem Pharmacol. (2008) 75:369–82. 10.1016/j.bcp.2007.08.024 17919456

[13] Riccardi C Nicoletti I. Analysis of apoptosis by propidium iodide staining and flow cytometry. Nat Protoc. (2006) 1:1458–61. 10.1038/nprot.2006.238 17406435

[14] Bradford MM. A rapid and sensitive method for the quantitation of microgram quantities of protein utilizing the principle of protein-dye binding. Anal Biochem. (1976) 72:248–54. 10.1016/0003-2697(76)90527-3 942051

[15] Sánchez-Aragó M Cuezva JM The bioenergetic signature of isogenic colon cancer cells predicts the cell death response to treatment with 3-bromopyruvate, iodoacetate or 5-fluorouracil. J Transl Med. (2011) 9:19 10.1186/1479-5876-9-19 21303518 PMC3045315

[16] Krysko DV Vanden Berghe T D'Herde K Vandenabeele P. Apoptosis and necrosis: detection, discrimination and phagocytosis. Methods. (2008) 44:205–21. 10.1016/j.ymeth.2007.12.001 18314051

[17] Vermes I Haanen C Reutelingsperger C. Flow cytometry of apoptotic cell death. J Immunol Methods. (2000) 243:167–90. 10.1016/S0022-1759(00)00233-7 10986414

[18] Newman DJ Cragg GM. Natural products as sources of new drugs over the 30 years from 1981 to 2010. J Nat Prod. (2012) 75:311–35. 10.1021/np200906s 22316239 PMC3721181

[19] Patil SD A recent review on anticancer herbal drugs. J drug Discov Ther. (2013) 1:77–84.

[20] Kheyar-Kraouche N da Silva AB Serra AT Bedjou F Bronze MR. Characterization by liquid chromatography–mass spectrometry and antioxidant activity of an ethanolic extract of *Inula viscosa* leaves. J Pharm Biomed Anal. (2018) 156:297–306. 10.1016/j.jpba.2018.04.047 29730339

[21] Benbacer L Merghoub N El Btaouri H Gmouh S Attaleb M Morjani H Antiproliferative effect and induction of apoptosis by *Inula viscosa* L. and *Retama monosperma* L. extracts in human cervical cancer cells. Top Cervical Cancer Advocacy Prev. (2012) 16:267–84. 10.5772/30025

[22] Merghoub N Btaouri H Benbacer L Gmouh S Trentesaux C Brassart B. *Inula viscosa* extracts induces telomere shortening and apoptosis in cancer cells and overcome drug resistance. Nutr Cancer. (2016) 68:131–43. 10.1080/01635581.2016.1115105 26771897

[23] Seca AML Grigore A Pinto DCGA Silva AMS. The genus Inula and their metabolites: from ethnopharmacological to medicinal uses. J Ethnopharmacol. (2014) 154:286–310. 10.1016/j.jep.2014.04.010 24754913

[24] Mahmoudi H Hosni K Zaouali W Amri I Zargouni H Hamida N Ben Comprehensive phytochemical analysis, antioxidant and antifungal activities of *Inula viscosa* aiton leaves. J Food Saf. (2016) 36:77–88. 10.1111/jfs.12215

[25] Maoz M Neeman I. Antimicrobial effects of aqueous plant extracts on the fungi *Microsporum canis* and *Trichophyton rubrum* and on three bacterial species. Lett Appl Microbiol. (1998) 26:61–3. 10.1046/j.1472-765X.1998.00277.x 9489036

[26] Ali-Shtayeh MS Yaghmour RM Faidi YR Salem K Al-Nuri MA. Antimicrobial activity of 20 plants used in folkloric medicine in the Palestinian area. J Ethnopharmacol. (1998) 60:265–71. 10.1016/S0378-8741(97)00153-0 9613839

[27] Brahmi-Chendouh N Piccolella S Crescente G Pacifico F Boulekbache L Hamri-Zeghichi S A nutraceutical extract from *Inula viscosa* leaves: UHPLC-HR-MS/MS based polyphenol profile, and antioxidant and cytotoxic activities. J Food Drug Anal. (2019). 10.1016/j.jfda.2018.11.006. [Epub ahead of print]. PMC9307043 31324285

[28] Gonzalez-Vallinas M Gonzalez-Castejon M Rodriguez-Casado A Ramirez de Molina A. Dietary phytochemicals in cancer prevention and therapy: a complementary approach with promising perspectives. Nutr Rev. (2013) 71:585–99. 10.1111/nure.12051 24032363

[29] Yin T-F Min Wang YQ Lin Y-M Wu D. Research progress on chemopreventive effects of phytochemicals on colorectal cancer and their mechanisms. World J Gastroenterol. (2016) 22:7058–68. 10.3748/wjg.v22.i31.7058 27610016 PMC4988307

[30] Ramos S. Effects of dietary flavonoids on apoptotic pathways related to cancer chemoprevention. J Nutr Biochem. (2007) 18:427–42. 10.1016/j.jnutbio.2006.11.004 17321735

[31] Kim M Miyamoto S Yasui Y Oyama T Murakami A Tanaka T. Zerumbone, a tropical ginger sesquiterpene, inhibits colon and lung carcinogenesis in mice. Int J Cancer. (2009) 124:264–71. 10.1002/ijc.23923t 19003968

[32] Prasad S Gupta SC Tyagi AK. Reactive oxygen species (ROS) and cancer: role of antioxidative nutraceuticals. Cancer Lett. (2017) 387:95–105. 10.1016/j.canlet.2016.03.042 27037062

[33] Bergman M Varshavsky L Gottlieb HE Grossman S. The antioxidant activity of aqueous spinach extract: chemical identification of active fractions. Phytochemistry. (2001) 58:143–52. 10.1016/S0031-9422(01)00137-6 11524124

[34] Khan N Afaq F Saleem M Ahmad N Mukhtar H. Targeting multiple signaling pathways by green tea polyphenol (-)-epigallocatechin-3-gallate. Cancer Res. (2006) 66:2500–5. 10.1158/0008-5472.CAN-05-3636 16510563

[35] Belayachi L Aceves-Luquero C Merghoub N Bakri Y Fernández de Mattos S Amzazi S Screening of North African medicinal plant extracts for cytotoxic activity against tumor cell lines. Eur J Med Plants. (2013) 3:310–32. 10.9734/EJMP/2013/3403

[36] Pal HC Sehar I Bhushan S Gupta BD Saxena AK. Activation of caspases and poly (ADP-ribose) polymerase cleavage to induce apoptosis in leukemia HL-60 cells by *Inula racemosa*. Toxicol Vitr. (2010) 24:1599–609. 10.1016/j.tiv.2010.06.007 20600805

[37] Russo P Malacarne D Falugi C Trombino S O'Connor PM. RPR-115135, a farnesyltransferase inhibitor, increases 5-FU-cytotoxicity in ten human colon cancer cell lines: role of p53. Int J Cancer. (2002) 100:266–75. 10.1002/ijc.10461 12115540

[38] Fischer M Quaas M Steiner L Engeland K. The p53-p21-DREAM-CDE/CHR pathway regulates G2/M cell cycle genes. Nucleic Acids Res. (2016) 44:164–74. 10.1093/nar/gkv927 26384566 PMC4705690

[39] Murakami Y Hayashi K Sekiya T. Detection of aberrations of the p53 alleles and the gene transcript in human tumor cell lines by single-strand conformation polymorphism analysis. Cancer Res. (1991) 51: 3356–61. 2054775

[40] Fulda S Debatin KM. Extrinsic versus intrinsic apoptosis pathways in anticancer chemotherapy. Oncogene. (2006) 25:4798–811. 10.1038/sj.onc.1209608 16892092

[41] Smulson ME Simbulan-Rosenthal CM Boulares AH Yakovlev A Stoica B Iyer S. Roles of poly(ADP-ribosyl)ation and PARP in apoptosis, DNA repair, genomic stability and functions of p53 and E2F-1. Adv Enzyme Regul. (2000) 40:183–215. 10.1016/S0065-2571(99)00024-2 10828352

[42] Lan Y-H Chiang J-H Huang W-W Lu C-C Chung J-G Wu T-S. Activations of both extrinsic and intrinsic pathways in HCT 116 human colorectal cancer cells contribute to apoptosis through p53-mediated ATM/Fas signaling by Emilia sonchifolia extract, a folklore medicinal plant. Evid Based Complement Altern Med. (2012) 2012:178178. 10.1155/2012/178178 22474491 PMC3303801

[43] Derry M Raina K Agarwal R Agarwal C. Differential effects of grape seed extract against human colorectal cancer cell lines: the intricate role of death receptors and mitochondria. Cancer Lett. (2012) 334:69–78. 10.1016/j.canlet.2012.12.015 23268334 PMC3622127

[44] Li M Song L-H Yue GG-L Lee JK-M Zhao L-M Li L. Bigelovin triggered apoptosis in colorectal cancer *in vitro* and *in vivo* via upregulating death receptor 5 and reactive oxidative species. Sci Rep. (2017) 7:42176. 10.1038/srep42176 28181527 PMC5299840

[45] Wang G-W Qin J-J Cheng X-R Shen Y-H Shan L Jin H-Z. *Inula sesquiterpenoids*: structural diversity, cytotoxicity and anti-tumor activity. Expert Opin Investig Drugs. (2014) 23:317–45. 10.1517/13543784.2014.868882 24387187

[46] Kamei H Koide T Kojimam T Hasegawa M Terabe K Umeda T. Flavonoid-mediated tumor growth suppression demonstrated by *in vivo* study. Cancer Biother Radiopharm. (1996) 11:193–6. 10.1089/cbr.1996.11.193 10851537

[47] Ren W Qiao Z Wang H Zhu L Zhang L. Flavonoids: promising anticancer agents. Med Res Rev. (2003) 23:519–34. 10.1002/med.10033 12710022

[48] de Jonge MJA Verweij J. Renal toxicities of chemotherapy. Semin Oncol. (2006) 33:68–73. 10.1053/j.seminoncol.2005.11.011 16473645

[49] Ramadori G Cameron S. Effects of systemic chemotherapy on the liver. Ann Hepatol. (2010) 9:133–43. 20526005

[50] Rubbia-Brandt L Audard V Sartoretti P Roth AD Brezault C Le Charpentier M. Severe hepatic sinusoidal obstruction associated with oxaliplatin-based chemotherapy in patients with metastatic colorectal cancer. Ann Oncol. (2004) 15:460–6. 10.1093/annonc/mdh095 14998849

[51] Dunn SR Qi Z Bottinger EP Breyer MD Sharma K. Utility of endogenous creatinine clearance as a measure of renal function in mice. Kidney Int. (2004) 65:1959–67. 10.1111/j.1523-1755.2004.00600.x 15086941

